# Impact of a nurse-led educational intervention on knowledge and adherence to hemodialysis in Kiambu County, Kenya: a quasi-experimental study

**DOI:** 10.3389/fpubh.2026.1869001

**Published:** 2026-06-24

**Authors:** Gabriel Njuguna Kilonzo, Elijah Mwangi Githinji, Drusilla Makworo, Ermias Mergia Terefe

**Affiliations:** 1 School of Nursing, Jomo Kenyatta University of Agriculture and Technology (JKUAT), Nairobi, Kenya; 2 Department of Pharmacology, Pharmacognosy, and Pharmaceutical Chemistry, School of Pharmacy and Health Sciences, United States International University-Africa, Nairobi, Kenya

**Keywords:** adherence, chronic kidney disease, hemodialysis, nurse-led intervention, patient education

## Abstract

**Background:**

Chronic kidney disease (CKD) affects approximately 13.4% of the global population, and many patients require maintenance hemodialysis (HD). However, adherence to the HD treatment regimen remains suboptimal and is associated with adverse clinical outcomes. This study assessed the effect of a nurse-led educational intervention on patients’ knowledge of and adherence to the HD treatment regimen.

**Methods:**

A quasi-experimental study involving 129 patients undergoing maintenance HD was conducted between March and June 2025 at three dialysis centers in Kenya. Participants were followed for three months following the implementation of a nurse-led education intervention. Data were collected using a modified End-Stage Renal Disease Adherence Questionnaire (ESRD-AQ) and a structured knowledge questionnaire. Statistical analyses included paired and independent samples t-tests, McNemar and chi-square tests, and effect size estimation using Cohen’s d.

**Results:**

In the intervention group, mean knowledge scores increased from 45.0% at baseline to 79.0% at endline, whereas only a modest increase was observed in the control group (43.0 to 46.0%). Adherence also improved significantly in the intervention group, with the proportion of participants achieving satisfactory adherence increasing from 18.5 to 69.6%, while no meaningful change was observed in the control group. Both within-group and between-group analyses demonstrated large intervention effects (Cohen’s *d* = 2.96 and 2.85, respectively).

**Conclusion:**

The nurse-led educational intervention was associated with significant improvements in knowledge and adherence to the HD treatment regimen among patients undergoing maintenance HD. These findings suggest that structured patient education may be an effective strategy for enhancing treatment adherence and improving patient outcomes in resource-limited settings.

## 1 Introduction

Chronic kidney disease (CKD) is a growing global public health concern, affecting approximately 13.4% of the world’s population and contributing substantially to morbidity and mortality (1, 2). The World Health Organization (WHO) ranked CKD as the tenth leading cause of death worldwide in 2020 and projects that it will become the fifth leading cause of mortality by 2040 (3, 4). The burden of CKD is disproportionately higher in low- and middle-income countries, particularly in Sub-Saharan Africa (SSA), where prevalence ranges between 10.1 and 15.8% and access to comprehensive renal care remains limited (1, 5, 6). In Kenya, more than 10,000 new CKD cases are diagnosed annually, with an estimated 4.8 million individuals projected to be living with kidney disease by 2030 (7–9).

Patients with advanced CKD require renal replacement therapy, including HD, peritoneal dialysis, or kidney transplantation, with HD being the most commonly utilized modality (10, 11). Maintenance HD requires strict adherence to a complex treatment regimen involving regular dialysis attendance, medication use, fluid restriction, and dietary modifications (12). However, adherence across these domains remains suboptimal. Globally, approximately 50% of patients on maintenance HD are non-adherent, with similar rates reported in Africa (13). In Kenya, non-adherence rates of up to 52.6% have been reported among patients receiving maintenance HD (7). Poor adherence is associated with increased morbidity, frequent hospitalizations, and mortality rates that are several-fold higher than those observed in the general population (10, 13, 14).

Effective self-management and patient education are widely recognized as key determinants of adherence to the HD treatment regimen. The Chronic Care Model emphasizes self-management support as a central component of high-quality chronic disease care and improved health outcomes (15–17). Despite this, structured and contextually adapted educational interventions for patients undergoing maintenance HD remain limited in SSA. Although international guidelines, such as those developed by Kidney Disease: Improving Global Outcomes (KDIGO), recommend comprehensive patient education, their direct application without adaptation to local sociocultural and healthcare system contexts may limit effectiveness (18–20).

Evidence from high- and middle-income countries indicates that nurse-led educational and behavioral interventions, particularly those integrating written materials, audiovisual tools, and structured follow-up, can improve adherence to dialysis attendance, medication use, and fluid and dietary restrictions, as well as selected clinical outcomes (21–23). However, the effectiveness of such interventions has not been adequately evaluated in Kenyan or other SSA dialysis settings.

At Avenue Hospital Thika, the largest dialysis center in Thika Sub-County, facility-level data indicate persistent challenges related to poor adherence, frequent hospital readmissions, and avoidable mortality among patients on maintenance HD. Currently, no structured nurse-led education program exists to systematically support adherence in this setting. Addressing this gap is essential for improving patient outcomes and developing scalable interventions for similar resource-constrained environments.

This study assessed adherence to the HD treatment regimen among patients on maintenance HD, identified factors associated with adherence, and evaluated the effectiveness of a nurse-led educational intervention in improving knowledge and adherence. The findings are intended to inform the development of structured, contextually appropriate patient education strategies aimed at improving treatment outcomes in Kenya and similar resource-constrained settings.

## 2 Methods

### 2.1 Study design and setting

This study employed a non-equivalent control group quasi-experimental design conducted between March and June 2025 in the renal units of Avenue Hospitals in Thika, Nairobi, and Kisumu, Kenya. Random allocation of facilities was not feasible due to operational and administrative considerations within the participating hospitals. Consequently, Avenue Hospital Thika was purposively selected as the intervention site because it had the largest dialysis unit, the highest patient volume, and the operational capacity required for the implementation and monitoring of the educational intervention. Avenue Hospitals Nairobi and Kisumu served as control sites and continued to provide routine standard care throughout the study period.

Because the intervention and control facilities were geographically separated and operated independently, the risk of contamination between study groups was minimized. Baseline socio-demographic and clinical characteristics were assessed before implementation to evaluate comparability between groups. Standardized eligibility criteria, data collection procedures, and outcome assessment tools were applied across all sites to minimize selection bias and measurement bias.

### 2.2 Study population

The study included adult outpatients with chronic kidney disease (CKD) undergoing maintenance HD for at least three months who were able to read and write, provided informed consent, and were present during the study period. Patients with acute kidney injury, those undergoing HD for less than three months, critically ill patients, individuals with cognitive impairment, and those unavailable during the study period were excluded.

A census sampling approach was adopted because the total accessible population of patients undergoing maintenance HD across the three participating facilities was relatively small (*N* = 170). Inclusion of all eligible patients was therefore considered feasible and preferable to sample-based estimation, as it maximized statistical power, minimized sampling error, and enhanced the representativeness of the target population.

### 2.3 Study variables

The independent variables included patient-related and therapy-related factors assessed at baseline. The primary outcome variable was adherence to the HD treatment regimen, measured at baseline and three months post-intervention, including dialysis attendance, medication use, dietary compliance, and fluid restriction. The intervention consisted of a structured nurse-led educational program designed to improve patients’ knowledge and adherence. Potential confounding variables included duration on HD, prior exposure to dialysis education, comorbid conditions, social support, and health literacy.

### 2.4 Theoretical framework

The educational intervention was guided by the Health Belief Model (HBM), which proposes that health behaviors are influenced by individuals’ perceptions of disease susceptibility, disease severity, perceived benefits of action, perceived barriers, self-efficacy, and cues to action. The HBM was selected because adherence to the HD treatment regimen requires sustained behavior change and active patient participation in self-management activities.

Intervention content was designed to address key HBM constructs. Perceived susceptibility and perceived severity were addressed through education on chronic kidney disease progression, treatment complications, and consequences of non-adherence. Perceived benefits were emphasized through discussions of improved health outcomes associated with adherence to dialysis schedules, medications, dietary recommendations, and fluid restrictions.

Perceived barriers were addressed by identifying common challenges experienced by participants and discussing practical strategies to overcome them. Self-efficacy was enhanced through skills-building activities, individualized guidance, and reinforcement of self-management behaviors. Cues to action were provided through educational materials, discussions, and ongoing interaction with healthcare providers during dialysis visits.

### 2.5 Study tools

Data were collected using a modified End-Stage Renal Disease Adherence Questionnaire (ESRD-AQ) and a structured self-administered questionnaire. The ESRD-AQ was adapted to improve contextual relevance and applicability within the Kenyan HD setting. Modifications included the incorporation of context-specific adherence barriers, such as treatment costs, transportation challenges, appointment scheduling, distance to dialysis centers, and availability of social support.

Content validity was established through expert review involving specialists in nephrology nursing, renal medicine, patient education, and research methodology. The adapted instrument underwent two rounds of expert evaluation to assess relevance, clarity, comprehensiveness, and cultural appropriateness. Recommendations from the review process were incorporated before pilot testing.

The revised questionnaire was subsequently pretested among patients undergoing maintenance HD at Kenol Hospital to evaluate clarity, acceptability, feasibility, and comprehension. Feedback obtained during pretesting informed minor wording modifications before final implementation.

Internal consistency reliability was assessed using Cronbach’s alpha coefficients, which demonstrated acceptable reliability across adherence domains, including dialysis attendance (*α* = 0.712), medication adherence (α = 0.700), fluid restriction (α = 0.871), and dietary adherence (α = 0.746).

The knowledge questionnaire was developed by the principal investigator based on a literature review of CKD, HD, and patient self-management. It assessed knowledge related to dialysis attendance, medication adherence, dietary and fluid restrictions, vascular access care, and complications of non-adherence. Content validity was established through expert review by nephrology nursing, research methodology, and patient education specialists, and the tool was pretested among maintenance HD patients to ensure clarity, relevance, and comprehensibility.

### 2.6 Study phases

The study was conducted in three phases. Phase one involved a baseline assessment of patients’ knowledge, adherence to the HD treatment regimen, and selected patient- and therapy-related factors. Phase two involved the development and implementation of a nurse-led educational intervention based on baseline findings, published evidence, international kidney care guidelines, expert recommendations from a Delphi validation process, and the HBM. The intervention was delivered over three months through four structured face-to-face sessions lasting 45–60 min each and conducted during routine dialysis visits.

Session one focused on CKD, disease progression, and the importance of adherence to prescribed dialysis schedules. Session two addressed medication adherence, including the purpose, benefits, and consequences of missed medications. Session three focused on dietary management and fluid restriction, emphasizing practical strategies for daily self-management. Session four addressed coping strategies, self-care behaviors, complication prevention, and long-term treatment engagement.

**Figure 1:**
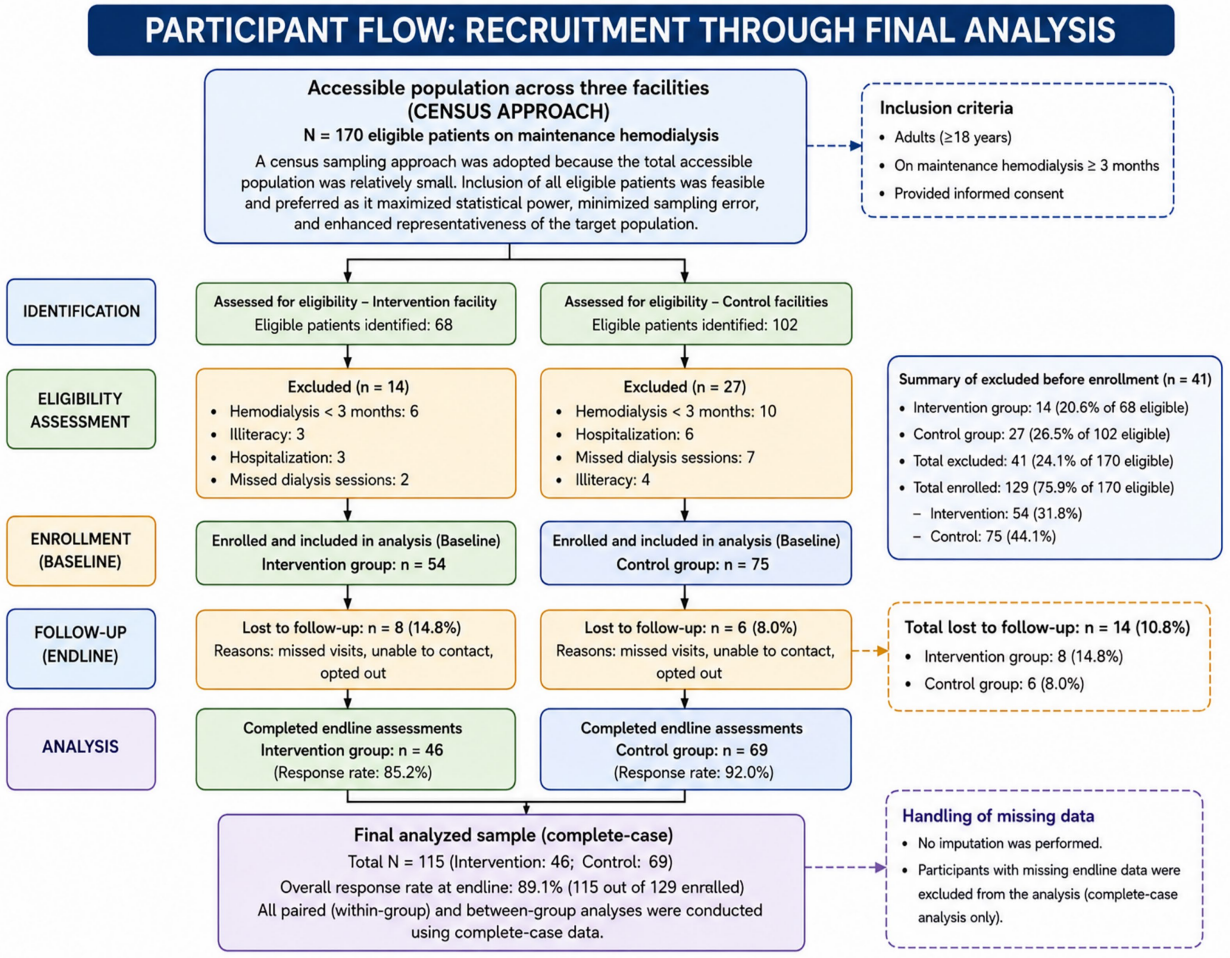
Participant flow diagram showing recruitment, allocation, follow-up, and analysis.

Educational content was delivered by trained nephrology nurses and research assistants using PowerPoint presentations, pamphlets, participant handbooks, and culturally adapted videos in English and Kiswahili. Participants were organized into small groups (≤8 participants) to promote interaction, peer support, and individualized learning. Participants received printed educational materials after each session and were encouraged to discuss adherence challenges throughout the intervention period. The control group received routine standard care. Phase three involved post-intervention assessment of knowledge and adherence in both groups three months after implementation (see Table 1; Figure 1).

**Table 1:** Components of the educational intervention.

Component	Description
Delivery mode	Face-to-face group education
Duration	3 months
Number of sessions	4
Session duration	45–60 min
Group size	≤8 participants
Educators	Trained nephrology nurses and research assistants
Educational materials	Pamphlets, PowerPoint presentations, and videos
Languages	Kiswahili and English
Reinforcement	Printed educational materials and ongoing discussion during dialysis visits
Control condition	Routine standard care

### 2.7 Study procedure

Data collection was conducted by the principal investigator and trained research assistants with prior nephrology nursing experience. Before data collection commenced, all research assistants underwent structured training facilitated by the principal investigator. The training covered study objectives, participant recruitment procedures, administration of study instruments, informed consent procedures, research ethics, confidentiality requirements, data quality assurance measures, and the standardization of data collection techniques. Practical demonstrations and mock data collection exercises were conducted to ensure consistency in questionnaire administration and minimize inter-observer variation across study sites. Ongoing supervision was provided throughout the study period to maintain protocol adherence and ensure data quality.

### 2.8 Data analysis

Data were analyzed using SPSS version 27. Descriptive statistics were used to summarize sociodemographic characteristics, knowledge, and adherence. Continuous variables were expressed as means and standard deviations, while categorical variables were presented as frequencies and percentages. The Kolmogorov–Smirnov test was used to assess normality. Adherence was measured using a Likert-based scoring system and categorized as satisfactory (≥70.0%) or unsatisfactory (<70.0%). Percentage scores were calculated by dividing individual scores by the maximum attainable score and multiplying by 100. Similarly, knowledge of the HD regimen was categorized as satisfactory (>80.0%) or unsatisfactory (<80.0%). Inferential analyses were conducted to evaluate intervention effectiveness using paired and independent samples t-tests, McNemar and chi-square tests, and effect size estimation, with statistical significance set at *p* < 0.05. Correlation analysis was not performed because intervention exposure was a binary categorical variable (intervention versus control). Comparative statistical methods were considered more appropriate for assessing differences in adherence outcomes between groups and overtime.

Cohen’s d was used to assess the effect size of the intervention. It was calculated by dividing the difference between group means by the pooled standard deviation. Effect sizes were interpreted according to Cohen’s criteria, whereby 0.2 represented a small effect, 0.5 a medium effect, and 0.8 a large effect.

To further evaluate intervention effectiveness, analysis of variance (ANOVA) was performed to assess differences in adherence score changes between the intervention and control groups. Effect size was estimated using eta squared (η^2^), with larger values indicating a greater proportion of variance explained by the intervention.

### 2.9 Ethical considerations

Ethical approval was obtained from the Nairobi Hospital Bioethics and Research Committee (NHBRC) (approval number: TNH/DMSR/ISERC/RP/001/24), and a research permit was granted by the National Commission for Science, Technology and Innovation (NACOSTI) (permit number: NACOSTI/P/24/33924). Permission to conduct the study was also obtained from Avenue Hospital administration. Written informed consent was obtained from all participants before enrollment. Participant confidentiality and anonymity were maintained throughout the study. The study was conducted in accordance with the principles of the Declaration of Helsinki.

## 3 Results

### 3.1 Participant retention and follow-up

A total of 129 participants were enrolled at baseline, including 54 in the intervention group and 75 in the control group. During follow-up, 14 participants were lost, including eight from the intervention group and six from the control group. Consequently, endline assessments were completed by 46 participants in the intervention group and 69 participants in the control group, resulting in an overall response rate of 89.1%. All paired analyses were conducted using complete-case analysis.

### 3.2 Demographic characteristics of participants

A total of 129 participants were enrolled in the study, including 54 in the intervention group and 75 in the control group. The mean age was 57.2 ± 13.2 years in the intervention group and 52.5 ± 13.8 years in the control group. Other sociodemographic characteristics are presented in Table 2.

**Table 2:** Socio-demographic characteristics of participants.

Variable	Intervention group (*n* = 54)	Control group (*n* = 75)
Age (years)	Mean = 57.2 ± 13.2	Mean = 52.5 ± 13.8
	Median = 57.5 (range: 28–85)	Median = 54 (range: 24–86)
Gender		
Male	39 (72.0%)	50 (66.7%)
Female	15 (27.8%)	25 (33.3%)
Education level		
Primary	17 (31.5%)	15 (20.0%)
Secondary	20 (37.0%)	20 (26.7%)
Tertiary	17 (31.5%)	40 (53.3%)

### 3.3 Knowledge of the HD treatment regimen

**Figure 2:**
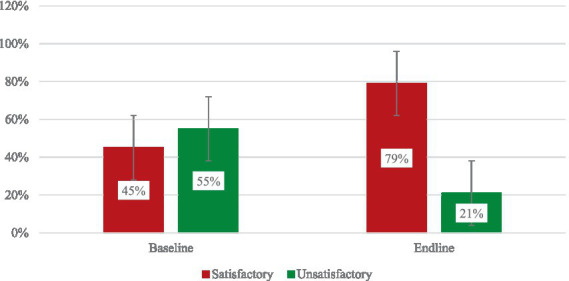
Mean knowledge scores (%) at baseline and endline among participants in the intervention group. Error bars represent 95% confidence intervals.

**Figure 3:**
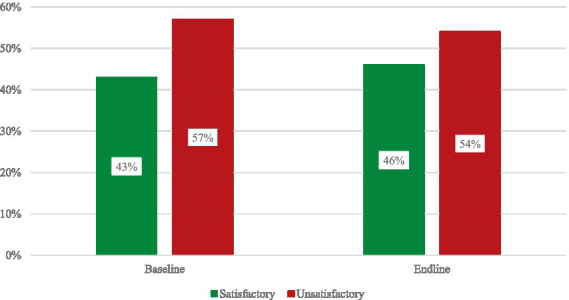
Mean knowledge scores (%) at baseline and endline among participants in the control group. Error bars represent 95% confidence intervals.

At baseline, the proportion of participants with satisfactory knowledge of the HD treatment regimen was 45.0% in the intervention group and 43.0% in the control group. At endline, the proportion increased to 79.0% in the intervention group, while only a slight increase was observed in the control group (46.0%). Changes in knowledge levels between baseline and endline differed between the two groups (Figures 2, 3).

### 3.4 Adherence to the HD treatment regimen

At baseline, adherence was highest for medication use (74.1% intervention; 74.7% control) and lowest for fluid (5.6% intervention; 4.0% control) and dietary restrictions (7.4% intervention; 17.3% control). Following the intervention, the intervention group showed marked improvements in all adherence domains: HD attendance increased from 33.3 to 54.3%, medication adherence from 74.1 to 89.1%, fluid adherence from 5.6 to 60.9%, and dietary adherence from 7.4 to 69.6%.

In contrast, only minimal changes were observed in the control group. Overall composite adherence improved substantially in the intervention group from 18.5 to 69.6%, while it declined in the control group from 26.7 to 17.3%, demonstrating the positive effect of the educational intervention on multiple components of treatment adherence. Detailed adherence outcomes are presented in Table 3.

**Table 3:** Adherence to components of the HD treatment regimen.

Component	Adherence status	Intervention baseline *n* (%)	Control baseline *n* (%)	Intervention endline *n* (%)	Control endline *n* (%)
HD frequency	Satisfactory	18 (33.3)	19 (25.3)	25 (54.3)	24 (34.8)
	Unsatisfactory	36 (66.7)	56 (74.7)	21 (45.7)	45 (65.2)
Medication	Satisfactory	40 (74.1)	56 (74.7)	41 (89.1)	53 (76.8)
	Unsatisfactory	14 (25.9)	19 (25.3)	5 (10.9)	16 (23.2)
Fluid	Satisfactory	3 (5.6)	3 (4.0)	28 (60.9)	5 (7.2)
	Unsatisfactory	51 (94.4)	72 (96.0)	18 (39.1)	64 (92.8)
Diet	Satisfactory	4 (7.4)	13 (17.3)	32 (69.6)	15 (21.7)
	Unsatisfactory	50 (92.6)	62 (82.7)	14 (30.4)	54 (78.3)
Composite adherence	Satisfactory	10 (18.5)	20 (26.7)	32 (69.6)	12 (17.3)
	Unsatisfactory	44 (81.5)	55 (73.3)	14 (30.4)	57 (82.6)

### 3.5 Within-group changes in adherence scores

Paired t-test analysis showed a significant increase in adherence scores in the intervention group, with the mean score increasing from 109.3 ± 4.65 at baseline to 138.39 ± 5.60 at endline (t (45) = −20.07, *p* < 0.001, Cohen’s d = 2.96). In the control group, a smaller increase was observed, with mean scores rising from 110.03 ± 3.45 to 111.64 ± 3.65 (t (68) = 3.34, *p* = 0.001, Cohen’s d = 0.40). Detailed results are presented in Table 4.

**Table 4:** Within-group changes in adherence scores (paired t-test).

Group	Baseline (mean ± SD)	Endline (mean ± SD)	Mean difference	95% CI	t (df)	*p*-value	Cohen’s d
Intervention	109.3 ± 4.65	138.39 ± 5.60	30.09	26.62 to 32.56	−20.07 (45)	<0.001	2.96
Control	110.03 ± 3.45	111.64 ± 3.65	1.61	0.27 to 3.34	3.34 (68)	0.001	0.40

### 3.6 Between-group comparison of adherence scores

At baseline, adherence scores were comparable between the intervention group (109.30 ± 11.35) and the control group (110.03 ± 12.09), with no statistically significant difference (t (127) = −0.35, *p* = 0.729, Cohen’s d = −0.06). At endline, the intervention group demonstrated significantly higher adherence scores (138.39 ± 5.60) compared with the control group (110.96 ± 11.55) (t (104.78) = 16.97, *p* < 0.001, Cohen’s d = 2.85). Detailed results are presented in Table 5.

**Table 5:** Between-group comparison of adherence scores (independent t-test).

Time point	Intervention group (mean ± SD)	Control group (mean ± SD)	Mean difference	t (df)	*p*-value	Cohen’s d
Baseline	109.30 ± 11.35	110.03 ± 12.09	−0.73	−0.35 (127)	0.729	−0.06
Endline	138.39 ± 5.60	110.96 ± 11.55	27.44	16.97 (104.78)	<0.001	2.85

### 3.7 Within-group changes in adherence proportions (McNemar’s test)

McNemar’s test showed a significant improvement in adherence within the intervention group, with the proportion of participants with satisfactory adherence increasing from 18.5% at baseline to 69.6% at endline (*p* < 0.001). In contrast, the control group showed no statistically significant change, with satisfactory adherence decreasing from 26.7 to 17.3% (*p* = 0.500). Detailed results are presented in Table 6.

**Table 6:** Within-group changes in adherence proportions (McNemar’s test).

Group	Adherence	Baseline *n* (%)	Endline *n* (%)	χ^2^	*p*-value
Intervention	Satisfactory	10 (18.5)	32 (69.6)	0.622	<0.001
	Unsatisfactory	44 (81.5)	14 (30.4)	-	-
Control	Satisfactory	20 (26.7)	12 (17.3)	0.040	0.500
	Unsatisfactory	55 (73.3)	57 (82.6)	-	-

### 3.8 Between-group differences in adherence proportions (chi-square test)

At baseline, there was no statistically significant difference in adherence between the intervention and control groups (χ^2^ = 1.17, *p* = 0.280). At endline, a statistically significant difference in adherence proportions was observed between the groups (χ^2^ = 17.01, *p* < 0.001). The odds of unsatisfactory adherence were lower in the intervention group compared with the control group (OR = 0.19). These results are presented in Table 7.

**Table 7:** Between-group differences in adherence proportions (Chi-square test).

Time point	Group	Satisfactory *n* (%)	Unsatisfactory *n* (%)	χ^2^	*p*-value	Odds ratio
Baseline	Intervention	10 (18.5)	44 (81.5)	1.17	0.280	1.60
	Control	20 (26.7)	55 (73.3)	-	-	-
Endline	Intervention	32 (69.6)	14 (30.4)	17.01	<0.001	0.19
	Control	12 (17.3)	57 (82.6)	-	-	-

### 3.9 Analysis of variance for adherence scores

Analysis of variance demonstrated a statistically significant intervention effect on adherence scores, *F* (1,113) = 523.51, *p* < 0.001, η^2^ = 0.822, indicating that approximately 82.2% of the variance in adherence score changes was attributable to the intervention. These results are presented in Table 8.

**Table 8:** Analysis of variance for adherence scores to the HD treatment regimen.

Source	Sum of squares	Df	Mean square	*F*-value	*p*-value	Partial η^2^	Observed power
Group (intervention vs. control)	22,009.58	1	22,009.58	523.51	<0.001	0.822	0.92
Error	4,750.80	113	42.04				
Corrected total	26,760.38	114					

## 4 Discussion

### 4.1 Principal findings

This study evaluated adherence to the HD treatment regimen and the effect of a nurse-led educational intervention on knowledge and adherence among patients receiving maintenance dialysis. At baseline, adherence was predominantly unsatisfactory, with more than three-quarters of participants in both study arms demonstrating poor composite adherence. Dietary restrictions and fluid management were the least adhered-to components. Following the implementation of the nurse-led educational intervention, significant improvements were observed in both knowledge and adherence in the intervention group, whereas only minimal changes occurred in the control group. The proportion of participants achieving satisfactory adherence increased substantially, and these improvements were supported by significant gains in mean adherence scores and large effect sizes. These findings suggest that structured nurse-led educational interventions can improve both patient knowledge and adherence to the HD treatment regimen.

### 4.2 Comparison with previous studies

The high prevalence of non-adherence observed at baseline is consistent with global evidence indicating persistently suboptimal adherence among patients undergoing maintenance HD, particularly with respect to dietary restrictions and fluid management (24–27). Similar findings have been reported across Sub-Saharan Africa, including studies conducted in Nigeria, Zimbabwe, and Rwanda, where non-adherence rates ranging from 49 to 73.5% have been documented (28–30).

The findings are also consistent with Kenyan studies reporting substantial challenges with adherence among patients receiving maintenance HD (7, 31). Collectively, these studies suggest that poor adherence remains a widespread challenge across dialysis settings and highlight the need for interventions that support patient self-management and engagement (39, 40).

The significant improvements in knowledge and adherence observed following the intervention are consistent with findings from studies conducted in Iran, India, and the United States, which have demonstrated that educational and nurse-led interventions can improve adherence to dialysis attendance, medication use, dietary recommendations, and fluid restrictions (32–35). The consistency of findings across diverse healthcare settings strengthens the evidence supporting structured educational interventions as an effective strategy for improving adherence among patients undergoing maintenance HD.

### 4.3 Interpretation of findings and theoretical implications

Several factors may explain the observed improvements in adherence. First, the intervention increased patients’ understanding of CKD, HD treatment requirements, and the consequences of non-adherence. The concurrent improvement in knowledge and adherence supports the role of patient education as a critical component of self-management in chronic disease care (15–17).

Second, the effectiveness of the intervention may be partly explained by its grounding in the HBM. Educational sessions were designed to influence participants’ perceptions of susceptibility to dialysis-related complications, the severity of poor adherence, the benefits of adherence, and strategies for overcoming perceived barriers. Reinforcement through educational materials and ongoing interaction with healthcare providers served as cues to action and strengthened self-efficacy. This finding is consistent with evidence suggesting that theory-based interventions are more effective in promoting sustained health behavior change than interventions lacking a theoretical foundation (36–38).

The observed improvements in adherence were accompanied by corresponding improvements in patient knowledge, suggesting a positive relationship between educational exposure and treatment adherence. Although formal correlation analysis was not performed, consistent improvements across both outcomes support the role of patient education in promoting adherence-related behaviors (32, 34).

The large effect sizes observed in this study should be interpreted cautiously. Although they indicate a substantial intervention effect, they may partly reflect low baseline levels of knowledge and adherence, the intensity of the intervention, and the use of multiple complementary educational approaches. The quasi-experimental design may also have contributed to the inflation of the effect size estimates. Nevertheless, the consistency of findings across multiple adherence outcomes supports the effectiveness of the intervention.

### 4.4 Clinical and policy implications

The findings have important implications for clinical practice and health policy. The results demonstrate that structured nurse-led educational interventions can be feasibly implemented within routine dialysis services and can significantly improve adherence-related behaviors among patients undergoing maintenance HD.

Healthcare facilities should consider integrating standardized patient education programs into routine renal care services. Such programs may improve self-management, reduce preventable complications, decrease hospital readmissions, and enhance quality of life among patients living with CKD (3, 18).

At the policy level, the incorporation of structured adherence education into national renal care guidelines may strengthen chronic disease management efforts and contribute to improved patient outcomes. Given the increasing burden of CKD in Kenya and across Sub-Saharan Africa, scalable and contextually adapted educational interventions represent a potentially cost-effective strategy for improving quality of care (1, 3).

### 4.5 Strengths

This study has several strengths. First, it employed a contextually adapted and theory-informed nurse-led educational intervention, which enhanced its relevance and feasibility for routine clinical practice. Second, the use of a structured and pretested instrument to assess adherence strengthened the reliability of the study measurements. Third, the inclusion of both knowledge and adherence outcomes, together with acceptable follow-up rates, enhanced the internal validity of the findings. Finally, the inclusion of both intervention and control groups strengthened the study design and improved the ability to assess the effect of the educational intervention on patient knowledge and adherence.

### 4.6 Limitations

This study has several limitations that should be considered when interpreting the findings. First, the study employed a quasi-experimental design without random allocation, which may have introduced selection bias and residual confounding. Second, adherence was assessed using self-reported questionnaires, making the findings susceptible to recall and social desirability bias. Third, the study was conducted in private tertiary healthcare facilities, which may limit the generalizability of the findings to public-sector dialysis settings and other healthcare contexts. Finally, post-intervention outcomes were assessed only once at three months following implementation of the intervention. Additional follow-up assessments at six and twelve months would have provided further insight into the sustainability of the observed improvements in knowledge and adherence over time.

## 5 Conclusion

Adherence to the DD treatment regimen was suboptimal at baseline among patients undergoing maintenance dialysis. The nurse-led educational intervention was associated with improvements in both knowledge and adherence at endline. These findings suggest that structured nurse-led education may be an effective approach for improving patient outcomes in resource-limited settings.

## 6 Recommendations

The findings of this study demonstrate that structured nurse-led educational interventions can significantly improve knowledge and adherence among patients undergoing maintenance HD. Integrating structured patient education into routine dialysis care may enhance self-management, reduce preventable complications, and improve patient outcomes. Healthcare institutions and policymakers should therefore consider incorporating standardized educational programs into renal care services to strengthen adherence support systems and improve the quality of care provided to patients with chronic kidney disease. Furthermore, future research should evaluate the long-term sustainability of intervention effects through extended follow-up periods and the use of randomized controlled study designs across multiple healthcare settings.

## Data Availability

The original contributions presented in the study are included in the article/supplementary material, further inquiries can be directed to the corresponding author.
